# GLSP and GLSP-derived triterpenes attenuate atherosclerosis and aortic calcification by stimulating ABCA1/G1-mediated macrophage cholesterol efflux and inactivating RUNX2-mediated VSMC osteogenesis

**DOI:** 10.7150/thno.80250

**Published:** 2023-02-21

**Authors:** Guobin Zheng, Yun Zhao, Zhenhao Li, Yunqing Hua, Jing Zhang, Yaodong Miao, Yang Guo, Lan Li, Jia Shi, Zhengwei Dong, Shu Yang, Guanwei Fan, Chuanrui Ma

**Affiliations:** 1 First Teaching Hospital of Tianjin University of Traditional Chinese Medicine, Tianjin, China; 2 National Clinical Research Center for Chinese Medicine Acupuncture and Moxibustion, Tianjin, China; 3 NHC Key Laboratory of Hormones and Development, Tianjin Key Laboratory of Metabolic Diseases, Chu Hsien-I Memorial Hospital & Tianjin Institute of Endocrinology, Tianjin Medical University, Tianjin 300134, China; 4 Department of Geriatrics, The Second Clinical Medical College, Jinan University (Shenzhen People's Hospital), Shenzhen, China; 5 Zhejiang ShouXianGu Botanical Drug Institute, Zhejiang Hangzhou 321200 China; 6 Second Affiliated Hospital of Tianjin University of Traditional Chinese Medicine, Tianjin, P. R. China

**Keywords:** GLSP, triterpenes, atherosclerosis, aortic calcification, cholesterol metabolism

## Abstract

**Background and Purpose:** Atherosclerosis is the main pathophysiological foundation of cardiovascular disease, which was caused by inflammation and lipid metabolism disorder, along with vascular calcification. Aortic calcification leads to reduced plaque stability and eventually causes plaque rupture which leads to cardiovascular events. Presently, the drug to treat aortic calcification remains not to be available. *Ganoderma lucidum* spore powder (GLSP) is from *Ganoderma lucidum* which is a Traditional Chinese Medicine with the homology of medicine and food. It has multiple pharmacological effects, but no research on aortic calcification during atherosclerosis was performed. This study investigated the effects of GLSP on atherosclerosis and aortic calcification and revealed the underlying mechanism.

**Methods:** *In vivo*, 8-week-aged male LDLR^-/-^ mice were fed a high-fat diet to induce atherosclerosis along with aortic calcification. Simultaneously, the mice were treated with GLSP at the first week of HFD feeding to determine the protection against early and advanced atherosclerosis. Subsequently, the mice tissues were collected to evaluate the effects of GLSP on atherosclerosis, and aortic calcification, and to reveal the underlying mechanism. *In vitro*, we determined the major components of GLSP triterpenes by HPLC, and subsequently assessed the protective effects of these main active components on lipid metabolism, inflammation, and calcification in RAW264.7 and HASMC cells.

**Results:** We observed GLSP attenuated plaque area and aortic calcification in the mice with early and advanced atherosclerosis. GLSP reduced the number of foam cells by improving ABCA1/G1-mediated cholesterol efflux in macrophages. In addition, GLSP protected against the aortic endothelium activation. Moreover, GLSP inhibited aortic calcification by inactivating RUNX2-mediated osteogenesis in HASMCs. Furthermore, we determined the major components of GLSP triterpenes, including Ganoderic acid A, Ganoderic acid B, Ganoderic acid C6, Ganoderic acid G, and Ganodermanontriol, and found that these triterpenes promoted ABCA1/G1-mediated cholesterol efflux and inhibited inflammation in macrophage, and inactivated RUNX2-mediated osteogenesis in VSMC.

**Conclusions:** This study demonstrates that GLSP attenuates atherosclerosis and aortic calcification by improving ABCA1/G1-mediated cholesterol efflux and inactivating RUNX2-mediated osteogenesis in LDLR^-/-^ mice. GLSP may be a potential drug candidate for the treatment of atherosclerosis and vascular calcification.

## Introduction

Cardiovascular diseases have high morbidity and mortality and have become the major cause of death worldwide 1. Atherosclerosis is the major pathogenesis of cardiovascular disease and is characterized by lipid-loaded lesions in the vascular wall. Lipids deposition in the vascular wall causes chronic inflammation, leading to the activation of vascular endothelium and secretion of a large number of adhesion molecules, which recruit circulating monocytes to infiltrate vascular endothelium and differentiate into macrophages. Subsequently, lipid-overloaded macrophages transfer into foam cells 2. The apoptosis of foam cells accelerates the formation of a necrotic core and promotes the development of lesions 3. Dysregulation of macrophage phenotypes is a major driver of atherosclerosis. In addition, macrophages with high plasticity polarize to pro-inflammatory M1 type and secrete a large number of inflammatory factors, which further accelerate atherosclerosis 4. As atherosclerosis develops, inflammation and lipid accumulation contribute to calcium deposition in the plaque, which leads to the generation of unstable plaque and cardiovascular events 5.

Foam cells are an important indicator for the development of atherosclerotic plaques. Promoting lipid efflux from macrophages is an important means to improve macrophage lipid homeostasis and inhibit foam cell formation 6. ATP-binding cassette transporters are members of the highly conserved membrane transporters superfamily, which play an important role in the transmembrane transport of lipids and lipoproteins in macrophages 7-9. ABCA1 and ABCG1 are important regulators of intracellular cholesterol homeostasis by transporting excess lipids from macrophages to HDL and apolipoprotein A-I, which are then transported to the liver in the form of cholesterol esters and metabolized to bile for excretion, namely cholesterol reverse transport 10. Upregulation of ABCA1 and ABCG1 expression can inhibit macrophage-derived foam cell formation and thereby effectively reduce the onset and progression of atherosclerosis 6.

Calcification is one of the factors leading to the instability of plaques 11. Vascular calcification is characterized by the deposition of inorganic calcium salts in the vascular walls, which leads to loss of elasticity and hemodynamic changes 12. Calcification in atherosclerotic plaques occurs in the vascular media and intima. Intimal calcification is triggered by inflammatory and cytokine stimuli, whereas medial calcification is driven by osteoblastic cells 13. As the calcification process proceeds, excessive production of microcalcifications combined with increased levels of inflammatory factors cause destabilized calcifications, which leads to plaque instability and even rupture 14. The mechanism underlying atherosclerotic calcification is mainly related to the transformation of vascular smooth muscle cells (VSMC) into osteoblast-like cells 15. The current clinical treatment for atherosclerosis mainly focuses on the strategy of lowering lipids, such as statins 16 and PCSK-9 inhibitors 17. Nevertheless, the hepatotoxicity and muscle toxicity of statins as well as the high cost of PCSK9 inhibitor treatment limit further utilization 17, 18. Moreover, although these drugs have been widely prescribed, there is still a large portion of patients experiencing cardiovascular disease. Most importantly, there is no effective treatment for vascular calcification during atherogenesis.

*Ganoderma lucidum* is a traditional natural medicine with the same origin as food and medicine. Modern pharmacological studies have shown that *Ganoderma lucidum* has multiple beneficial effects, such as lipid-lowering, anti-inflammation, anti-oxidative stress, and anti-apoptosis effects 19-22. *Ganoderma lucidum* spore powder (GLSP) is the germ cells from *Ganoderma lucidum*, which contains the biological components of *Ganoderma lucidum* 23. The above studies showed that GLSP has antiatherogenic potential. However, the protective effects of GLSP on atherosclerosis and vascular calcification as well as the underlying mechanism remains unknown. We investigated the antiatherogenic effects and mechanism of GLSP in LDLR^-/-^ mice with early or advanced atherosclerosis. This study revealed that GLSP can attenuate atherosclerosis and aortic calcification by reducing inflammation and apoptosis as well as VSMC osteogenesis. Subsequently, we detected the major components of GLSP by HPLC and performed the *in vitro* experiments to determine the protective effects of the triterpenoid content of GLSP on foam cell formation and inflammation in macrophages as well as calcification in VSMCs. Mechanistically, GLSP inhibited foam cell formation and aortic calcification by upregulating ABCA1/G1-mediated cholesterol efflux and inactivating RUNX2-mediated VSMC osteogenesis, respectively. Collectively, GLSP may be a new strategy for the treatment of atherosclerosis with calcification.

## Results

### GLSP attenuated early atherosclerosis and reduced plaque vulnerability in LDLR^-/-^ mice

**Figure 1:**
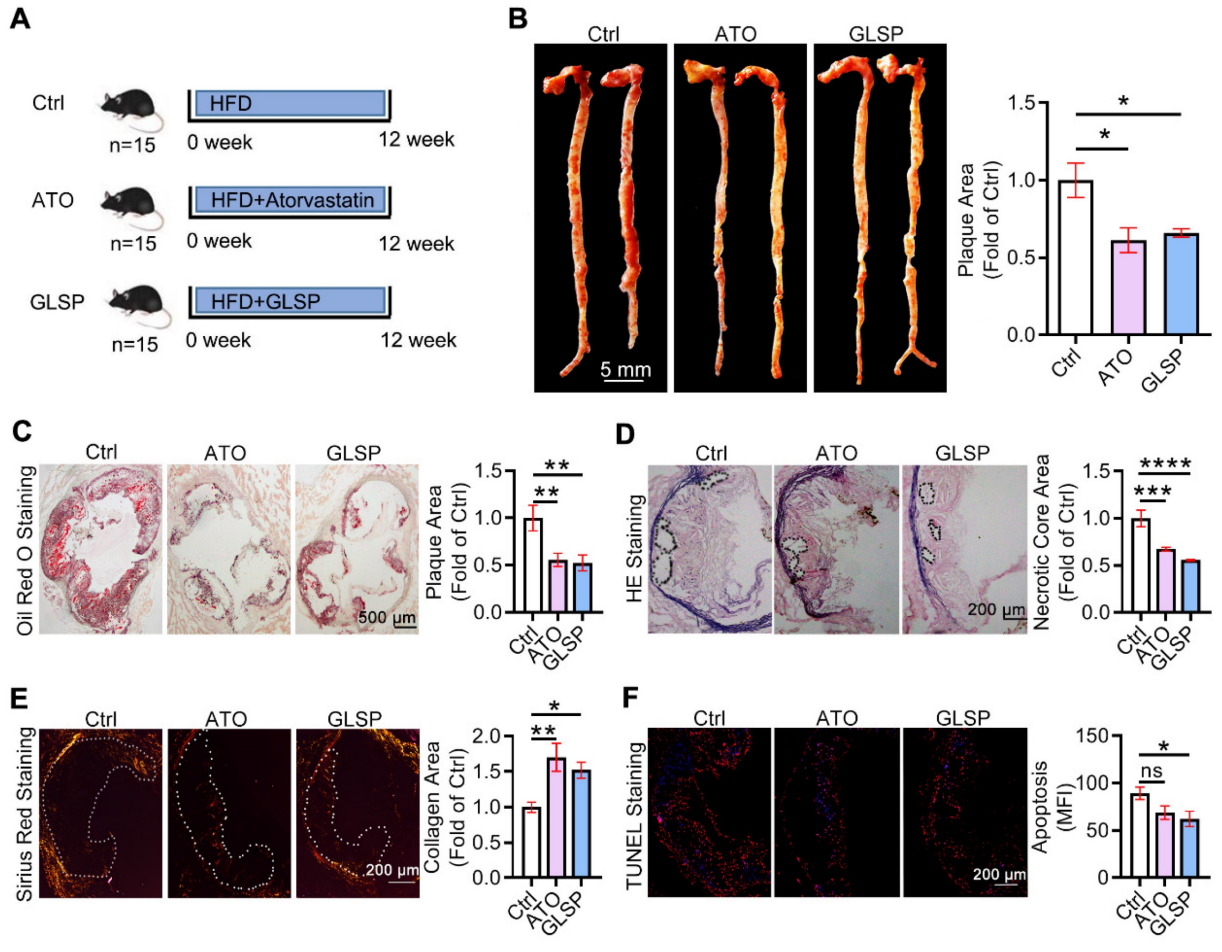
** The effects of GLSP on early atherosclerosis development in LDLR^-/-^ mice.** (**A**) Experiment design: male LDLR^-/-^ mice (8 weeks old) were randomly divided into 3 groups (15/group) and received the following treatment for 12 weeks: Ctrl group received HFD (High-fat diet), ATO group received HFD containing atorvastatin (4 mg/kg/day) and GLSP group received HFD containing GLSP (GLSP, 1400 mg/kg/day). (**B, C**) The lesions of *en face* aortas and aortic root cross-sections were determined by oil red O staining and analyzed quantitatively. (**D-F**) The necrotic core size, collagen content, and apoptosis level were determined by HE staining, Sirius red staining, and TUNEL staining respectively, and then the positive area was analyzed quantitatively. *P<0.05, **P<0.01, ***P<0.001, ****P<0.0001 compared with the Ctrl group. All experiments are presented as mean ± SEM.

To study whether GLSP could attenuate early atherosclerosis, 8-week-aged LDLR^-/-^ male mice were used to induce the early atherosclerosis model by feeding with HFD for 12 weeks, namely the Ctrl group. The other two groups were treated with atorvastatin (ATO) or GLSP, respectively, and the ATO group was indicated as the positive control (Figure 1A). In this study, the body weight of mice with early atherosclerosis showed no significance after the treatment of GLSP (Figure S1A-B). In addition, ultrasound data showed that cardiac function is not affected in mice with early atherosclerosis after administration of GLSP (Figure S2A). Subsequently, we examined the plaque area in the whole aorta and the aortic root. The oil red O staining revealed that the plaque area in the GLSP group was significantly reduced compared to the Ctrl group (Figure 1B-C). Increasing apoptosis within the plaque can contribute to the formation of a necrotic core during the development of atherosclerosis 24. The collagen-rich fibrous cap on the outside of the plaque becomes progressively thinner and leads to plaque instability 25. Thus, we examined the necrotic core, collagen content, and apoptotic levels of the plaque by HE, Sirius red, and TUNEL staining respectively (Figure 1D-F). After GLSP treatment, the area of the necrotic core within the plaque was significantly reduced, the collagen content was increased and the level of apoptosis was remarkably decreased. Taken together, GLSP markedly alleviated early atherosclerosis in LDLR^-/-^ mice and enhanced plaque stability, and this is achieved by reducing lipid deposition, apoptosis, shrinking the necrotic core, and increasing the collagen content in the plaque.

### GLSP regressed advanced atherosclerosis and enhanced the plaque stability in LDLR^-/-^ mice

**Figure 2:**
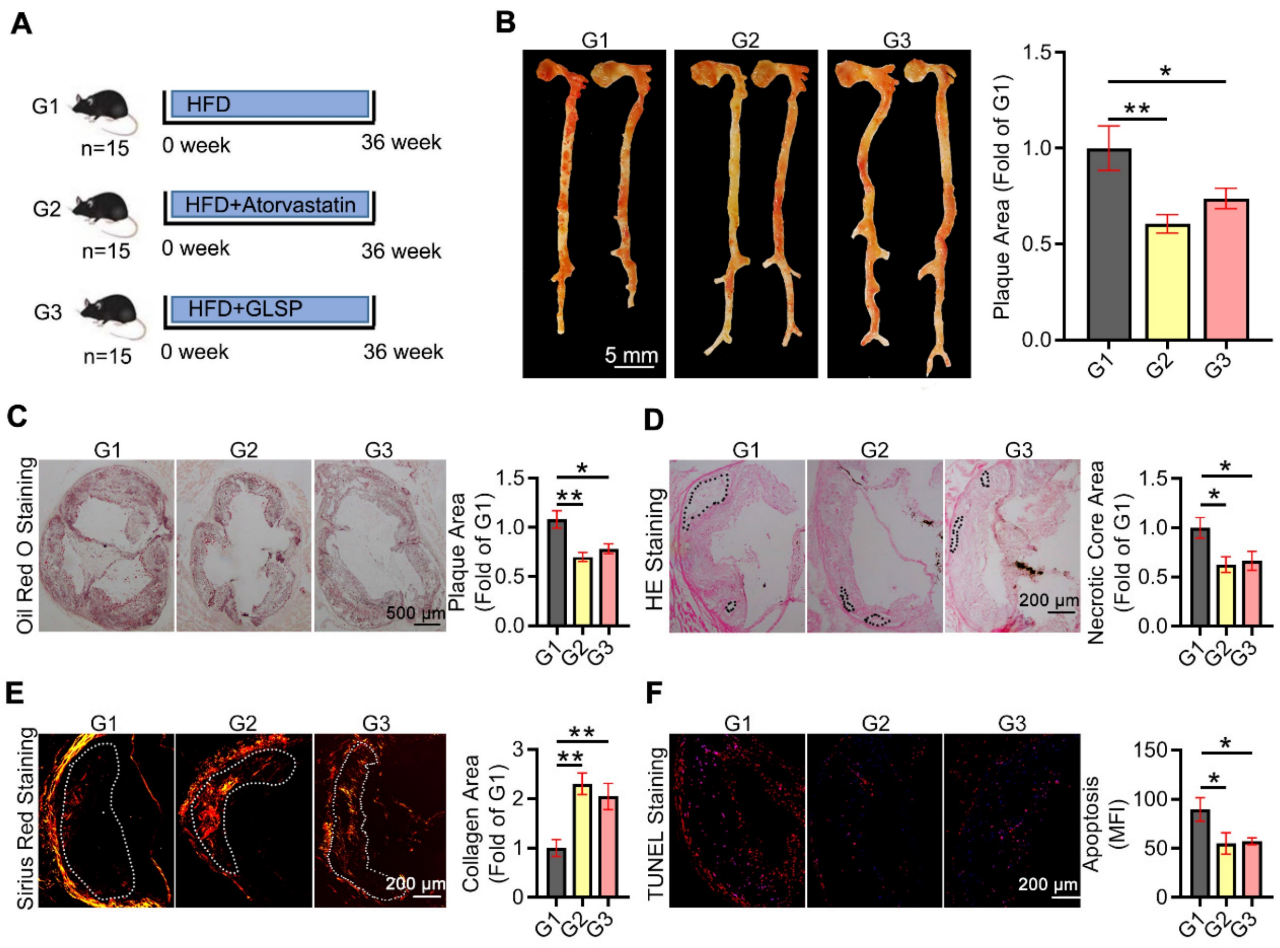
** The protective effects of GLSP on advanced atherosclerosis.** (**A**) Advanced atherosclerosis was induced by 36 weeks HFD fed. (**B, C**) Oil red O staining of the whole aorta and aortic root cross-sections and statistical analysis of plaque area in mice with advanced atherosclerosis. (**D**) Histopathological changes in the aortic root of advanced atherosclerosis mice were detected by HE staining. (**E**) Sirius red staining of aortic root cross-sections. (**F**) The results of TUNEL staining on aortic root sections. *P<0.05, **P<0.01 compared with the G1 group. All experiments are presented as mean ± SEM.

Advanced atherosclerotic plaque is prone to rupture and thereby leads to a cardiovascular event. Therefore, we further determined the ameliorative effect of GLSP on advanced atherosclerosis. The advanced atherosclerosis model was induced by 36 weeks of HFD treatment in the presence of ATO or GLSP (Figure 2A). Cardiac ultrasound results and the body weight of mice with advanced atherosclerosis showed no significant changes after GLSP administration (Figure S1C, Figure S2B). Intriguingly, GLSP decreased the area of advanced plaques with necrotic cores (Figure 2B-D), indicating that GLSP can repress advanced atherosclerosis. In addition, GLSP reduced the level of apoptosis, and increased the collagen content in the plaque (Figure 2E-F), suggesting that GLSP reduced plaque vulnerability. Collectively, these data suggest that GLSP regresses advanced atherosclerosis and enhances plaque stability in LDLR^-/-^ mice.

### GLSP inhibited the inflammation in both early and advanced atherosclerosis mice

**Figure 3:**
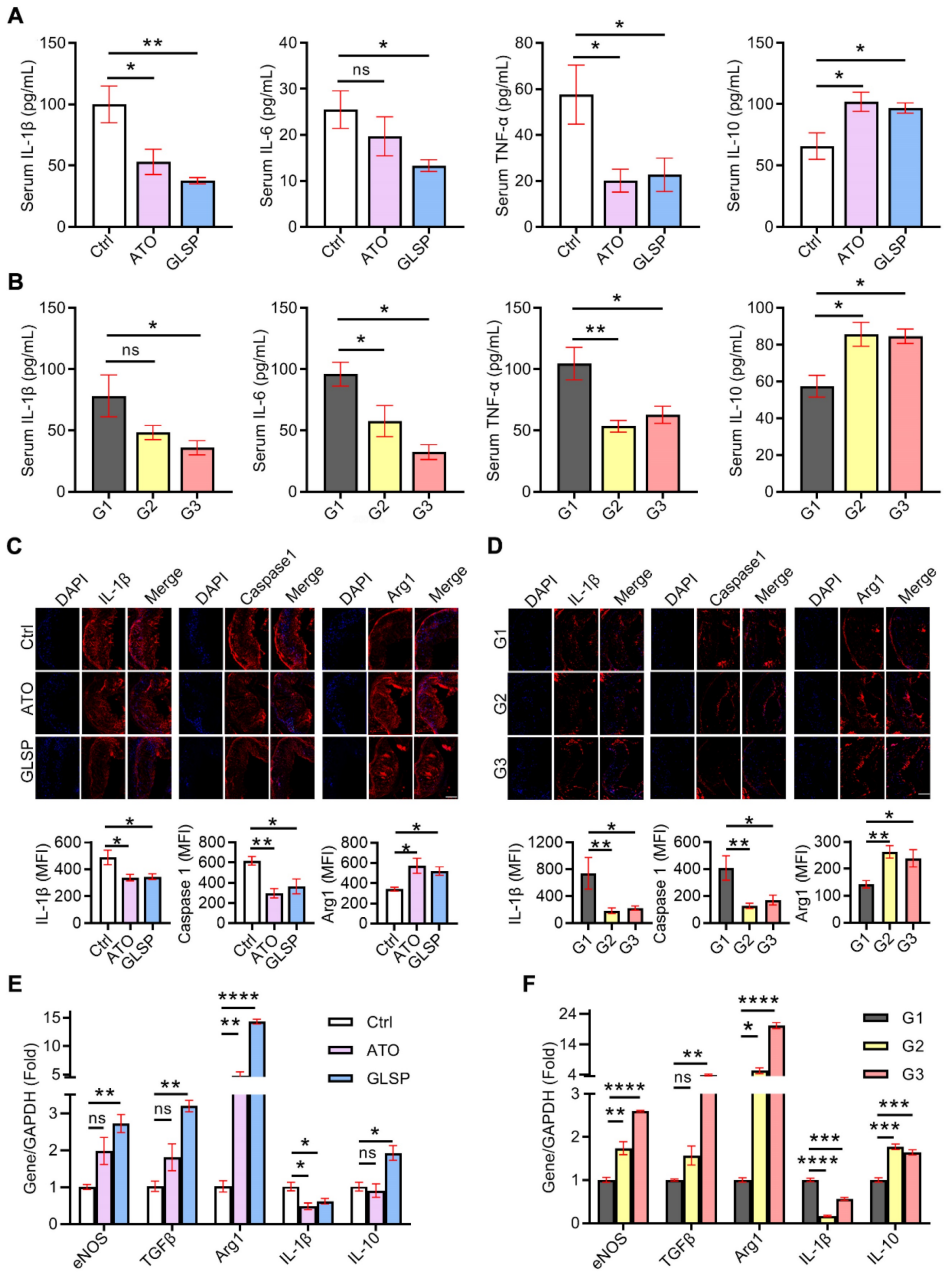
** GLSP reduced inflammation in early and advanced atherosclerosis mice.** (**A, B**) Effects of GLSP on serum levels of IL-1β, IL-6, TNF-α and IL-10 in mice with early and advanced atherosclerosis, *P<0.05, **P<0.01 compared with the Ctrl or G1 group. (**C, D**) Serial frozen sections of the aortic root in mice with early and advanced atherosclerosis were used for the immunofluorescence staining of IL-1 β, caspase1, and Arg1, *P<0.05, **P<0.01 compared with the Ctrl or G1 group. (**E, F**) The effects of GLSP on the expression of inflammatory factors in peritoneal macrophages of early and advanced atherosclerosis mice, *P<0.05, **P<0.01, ***P<0.001, ****P<0.0001 compared with the Ctrl or G1 group. All experiments are presented as mean ± SEM.

Inflammation is involved in all stages of atherosclerosis development. In the early stage of atherosclerosis, modified lipids activated inflammatory cells in the endothelium, secreting chemokines as well as adhesion factors to stimulate further inflammation. Subsequently, macrophages differentiated from monocytes also secreted inflammatory factors, and collectively these factors contributed to the development of atherosclerosis 26. Therefore, to determine the inhibitory effects of GLSP on inflammation in atherosclerosis, we first examined the serum levels of inflammatory factors in mice with early and advanced atherosclerosis, respectively. The serum IL-1β, IL-6, and TNF-α levels were significantly reduced, whereas IL-10 was greatly increased in mice with early and advanced atherosclerosis after GLSP treatment (Figure 3A-B). Next, immunofluorescence staining showed that intraplaque IL-1β and caspase1 levels were significantly reduced whereas Arg1 levels were significantly increased in early and advanced atherosclerosis after GLSP administration (Figure 3C-D). Macrophage-mediated inflammatory responses play a key role in atherogenesis. We extracted peritoneal macrophages from mice with early or advanced atherosclerosis and detected the expression levels of inflammatory factors by qRT-PCR. The level of the pro-inflammatory factor IL-1β was significantly decreased and the levels of anti-inflammatory factors eNOS, TGFβ, Arg1, and IL-10 were significantly increased after GLSP treatment in early and advanced atherosclerosis (Figure 3E-F). Collectively, these results suggest that GLSP can reduce inflammation, by which alleviating atherosclerosis.

### GLSP attenuated endothelium injury and reduced oxidative stress

**Figure 4:**
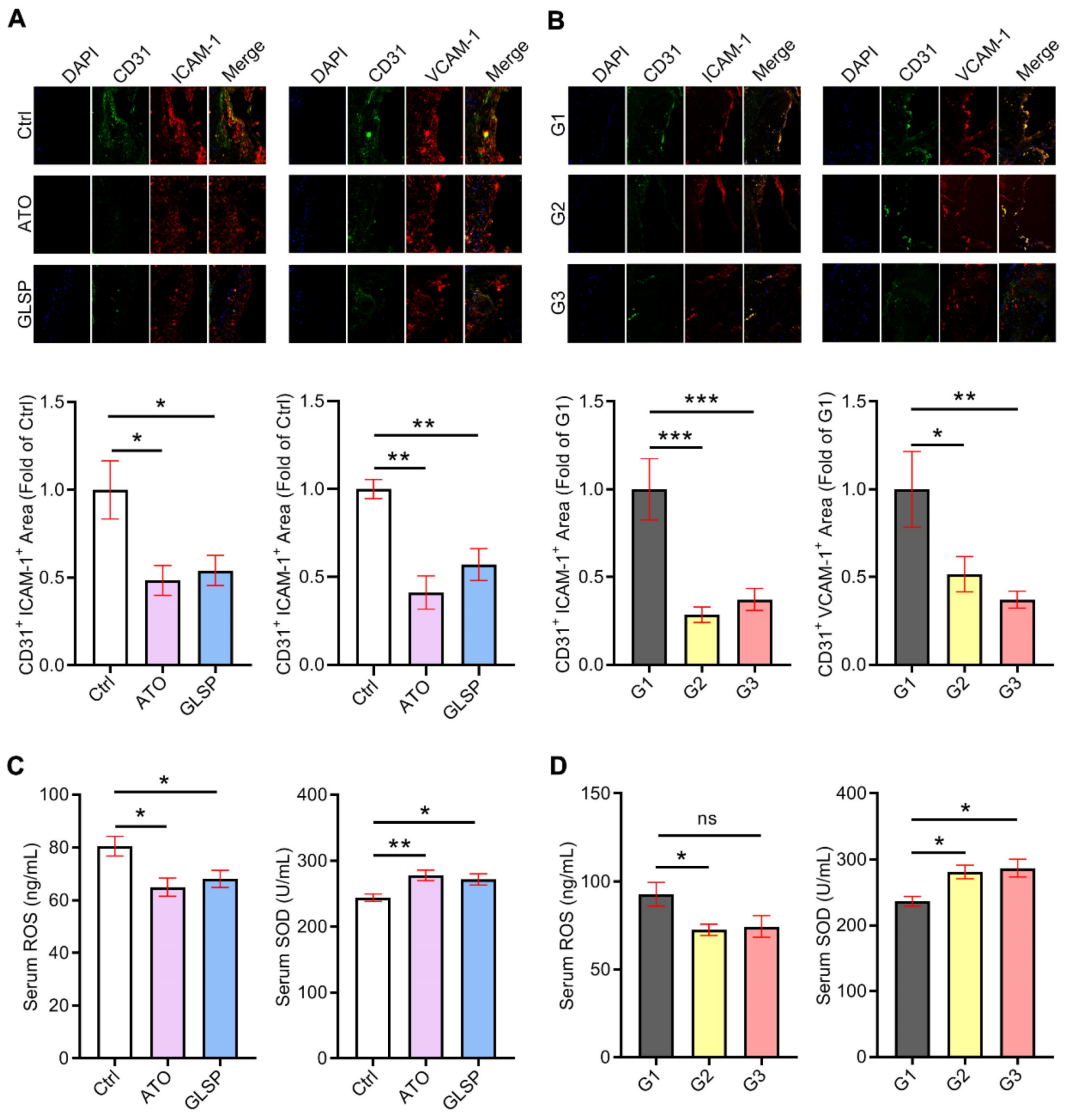
** The inhibitory effects of GLSP on endothelium injury and oxidant stress.** (**A, B**) Serial frozen sections of the aortic root in mice with early and advanced atherosclerosis were used for co-localization of immunofluorescence staining of CD31 and ICAM-1 or VCAM-1, *P<0.05, **P<0.01, ***P<0.001 compared with the Ctrl group or the G1 group. (**C**, **D**) Serum ROS and SOD levels in early or advanced atherosclerosis mice after GLSP administration, *P<0.05, **P<0.01 compared with the Ctrl or G1 group. All experiments are presented as mean ± SEM.

In addition to the inflammatory response, oxidative stress is considered another essential cause of atherosclerosis development. Large clusters of reactive oxygen species lead to the oxidation of lipids on the vascular endothelial cell membrane, resulting in endothelial dysfunction and monocyte adhesion. Therefore, we conducted an assay to evaluate the ability of GLSP to reduce endothelial damage and oxidative stress. We performed co-localization of immunofluorescence staining of CD31 and ICAM-1 or VCAM-1 in the aortic root sections of two stages of atherosclerosis. The results indicated that either atorvastatin or GLSP significantly reduced the levels of ICAM-1 and VCAM-1 in early as well as advanced plaques (Figure 4A-B), suggesting that GLSP could inhibit endothelial injury. In addition, to investigate the improvement of GLSP on oxidative stress, we examined the serum levels of ROS as well as SOD in mice. The results revealed that GLSP significantly increased serum SOD levels whereas decreased the serum ROS level in mice with early or advanced atherosclerosis (Figure 4C-D). Taken together, GLSP can reduce endothelial damage and oxidative stress, by which reducing the monocyte recruitment to the endothelium.

### GLSP inhibited macrophage-derived foam cell formation by improving ABCA1/G1-mediated cholesterol efflux

**Figure 5:**
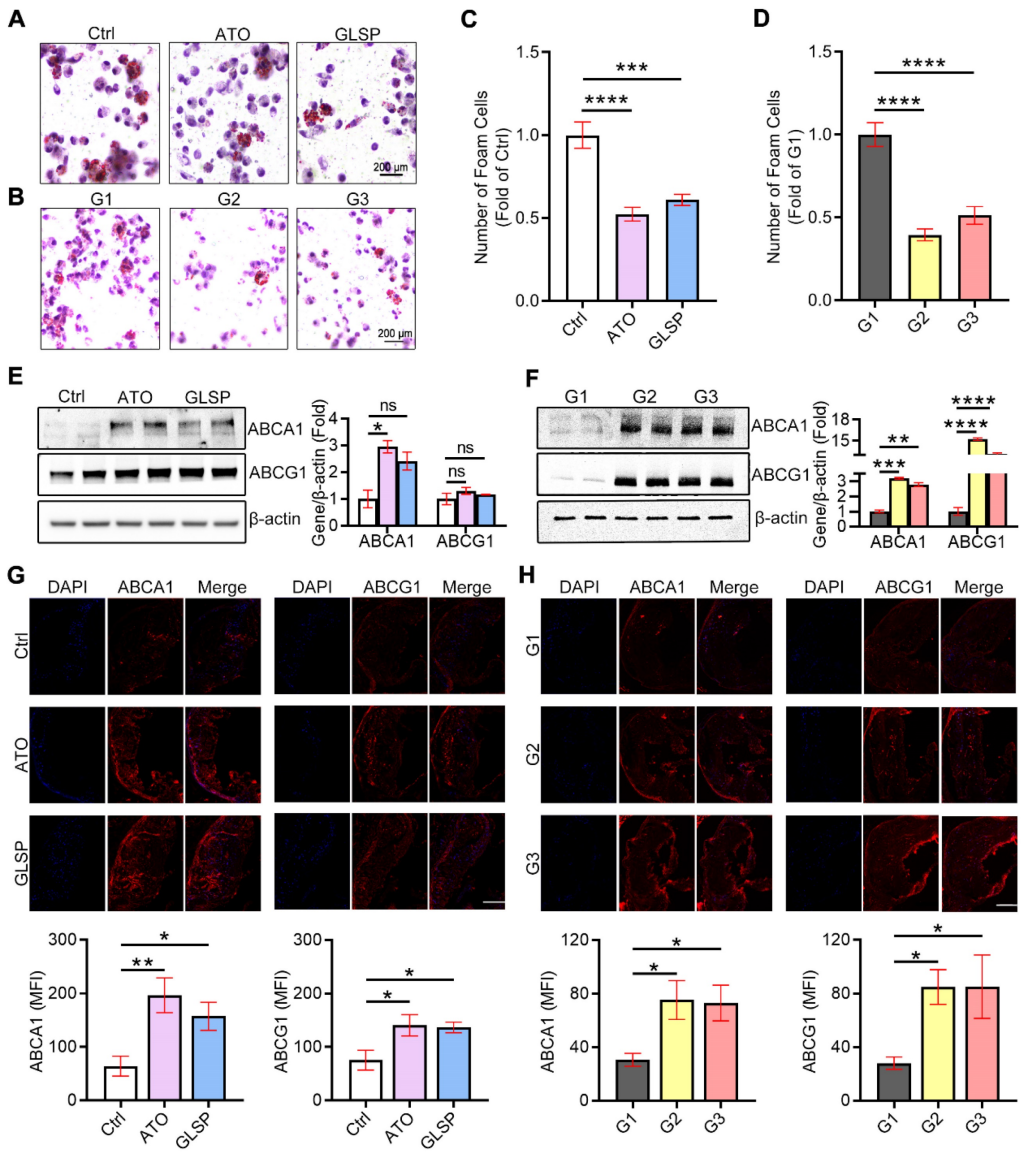
** Regulation of lipid metabolism in peritoneal macrophages by GLSP.** (**A-D**) Oil red O staining of peritoneal macrophages and statistical analysis of foam cells in early and advanced atherosclerosis mice, ***P<0.001, ****P<0.0001 compared with the Ctrl or G1 group. (**E, F**) Results of western blot assays for ABCA1 and ABCG1 in peritoneal macrophages from early and advanced atherosclerosis mice and quantitative statistical analysis, *P<0.05, **P<0.01, ***P<0.001, ****P<0.0001 compared with the Ctrl or G1 group. (**G**, **H**) Serial frozen sections of the aortic root in mice with early and advanced atherosclerosis were used for immunofluorescence staining of ABCA1 and ABCG1 *P<0.05, **P<0.01 compared with the Ctrl or G1 group. All experiments are presented as mean ± SEM.

Macrophage-derived foam cells are a major part of atherosclerosis plaque, which is inextricably linked to cellular lipids and especially cholesterol deposition 27, 28. To further explore the effects of GLSP on cholesterol homeostasis and foam cell formation, we extracted peritoneal macrophages in mice with early and advanced atherosclerosis and stained them with oil red O solution. The results showed that GLSP could significantly reduce lipid accumulation in macrophages (Figure 5A-D), which suggested that GLSP inhibited macrophage-derived foam cell formation. Subsequently, we verified the mechanism of GLSP in the regulation of cholesterol efflux by western blot assay, and the results showed that GLSP promoted the expression of ABCA1 and ABCG1 in peritoneal macrophages from the mice with early and advanced atherosclerosis (Figure 5E-F), which was further supported by the results of immunofluorescence staining of ABCA1 and ABCG1 in aortic root cross-sections (Figure 5G-H). Moreover, the qRT-PCR results showed that GLSP significantly upregulated genes related to lipid catabolism, and simultaneously downregulated genes related to cholesterol synthesis and lipid synthesis in peritoneal macrophages from the mice with early and advanced atherosclerosis (Figure S4A-B), which suggested that GLSP improved lipid metabolism in macrophages by promoting cholesterol efflux and lipid catabolism, as well as inhibiting lipid synthesis. In summary, GLSP can reduce lipid accumulation in macrophages by promoting cholesterol efflux and lipid catabolism as well as reducing lipid synthesis, which inhibits the transformation of macrophages into foam cells and plaque formation.

### GLSP improved the lipid metabolism in the liver under the condition of atherosclerotic dyslipidemia

**Figure 6:**
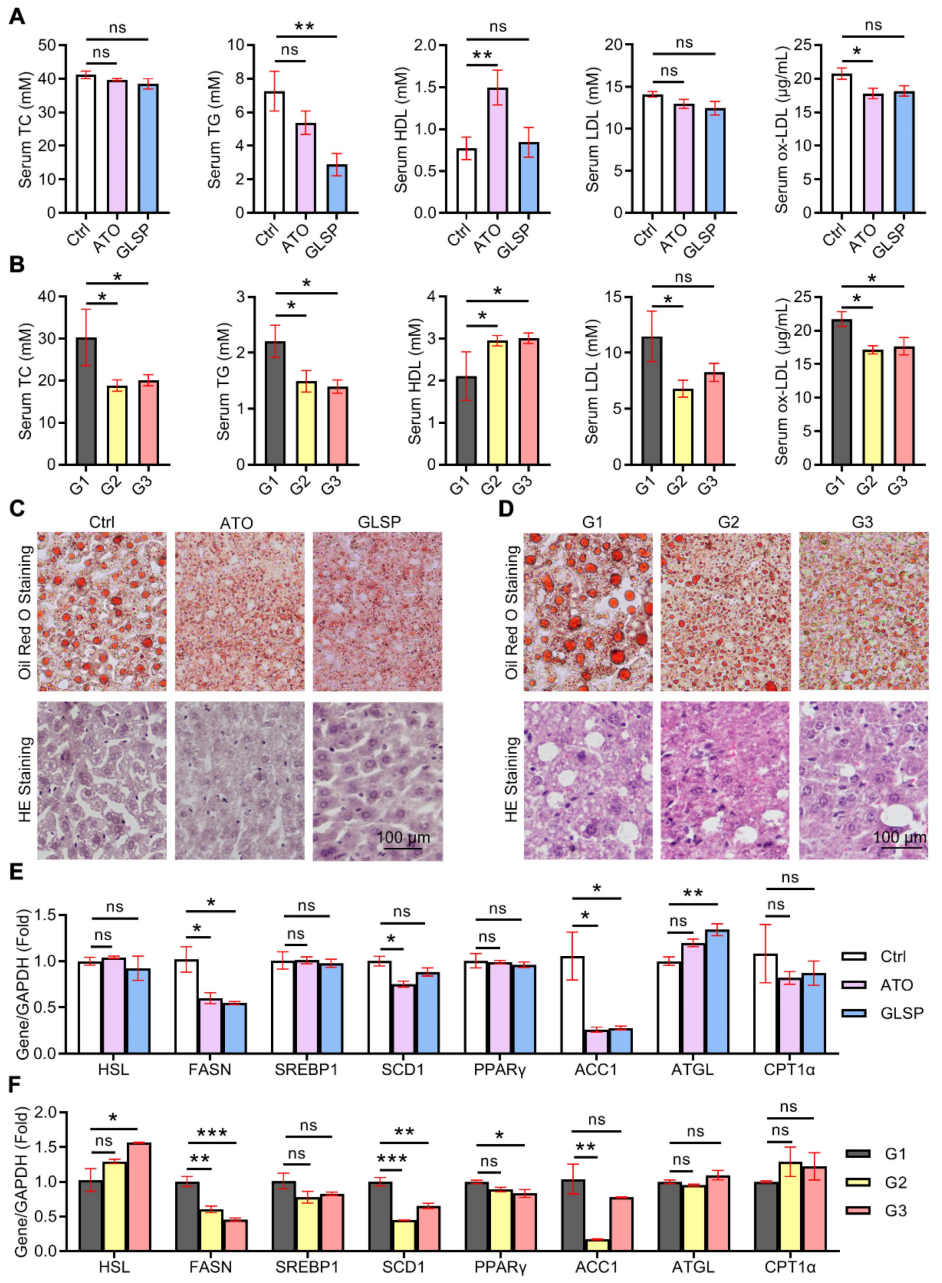
** GLSP reduced hepatic lipid deposition by regulating fatty acid metabolism.** (**A, B**) Effect of GLSP on serum lipids in mice with early and advanced LDLR^-/-^ mice. *P<0.05, **P<0.01, compared with the Ctrl or G1 group. (**C, D**) Oil red O staining and HE staining of the liver in early and advanced atherosclerosis mice. (**E, F**) Effects of GLSP on the expression of lipid metabolism-related genes in peritoneal macrophages from early and advanced atherosclerosis mice. *P<0.05, **P<0.01, ***P<0.001 compared with the Ctrl or G1 group. All experiments are presented as mean ± SEM.

Lipid metabolism has a crucial role in the pathogenesis of both NAFLD and atherosclerosis. Serum lipids were examined in early and advanced LDLR^-/-^ mice, and the results showed that GLSP significantly downregulated TG in the early model. GLSP significantly downregulated TC, TG, and ox-LDL, and upregulated HDL in the advanced model (Figure 6A-B). Abnormal hepatic lipid metabolism not only leads to NAFLD but also drives the development of atherosclerotic dyslipidemia 29, 30. To investigate the effect of GLSP on hepatic lipid metabolism, we measured the weight of the liver and subsequently gained the hepatosomatic index (liver weight /body weight, LW/BW). The results showed that the liver weight and hepatosomatic index of mice treated with GLSP tended to decrease without significance (Figure S1B, D). In addition, oil red O and HE staining were performed to evaluate the hepatic lipid accumulation. The staining results showed that the liver lipid accumulation and histopathological changes were significantly improved under GLSP treatment in mice fed with an HFD (Figure 6C, D). Furthermore, we extracted RNA from mouse livers and examined the expression of genes related to hepatic lipid synthesis and catabolism. The results of early atherosclerosis mice showed that GLSP was able to downregulate fatty acid synthesis-related genes (FASN, ACC1) and upregulate fatty acid catabolism-related genes (ATGL) (Figure 6E). In the results of advanced atherosclerosis mice, GLSP was able to downregulate fatty acid synthesis-related genes (FASN, PPARγ, SCD1) and upregulate fatty acid catabolism-related genes (HSL) (Figure 6F). Taken together, GLSP can improve hepatic lipid metabolism by reducing fatty acid synthesis and increasing fatty acid catabolism.

### GLSP reduced aortic calcification by inactivating RUNX2 signaling

**Figure 7:**
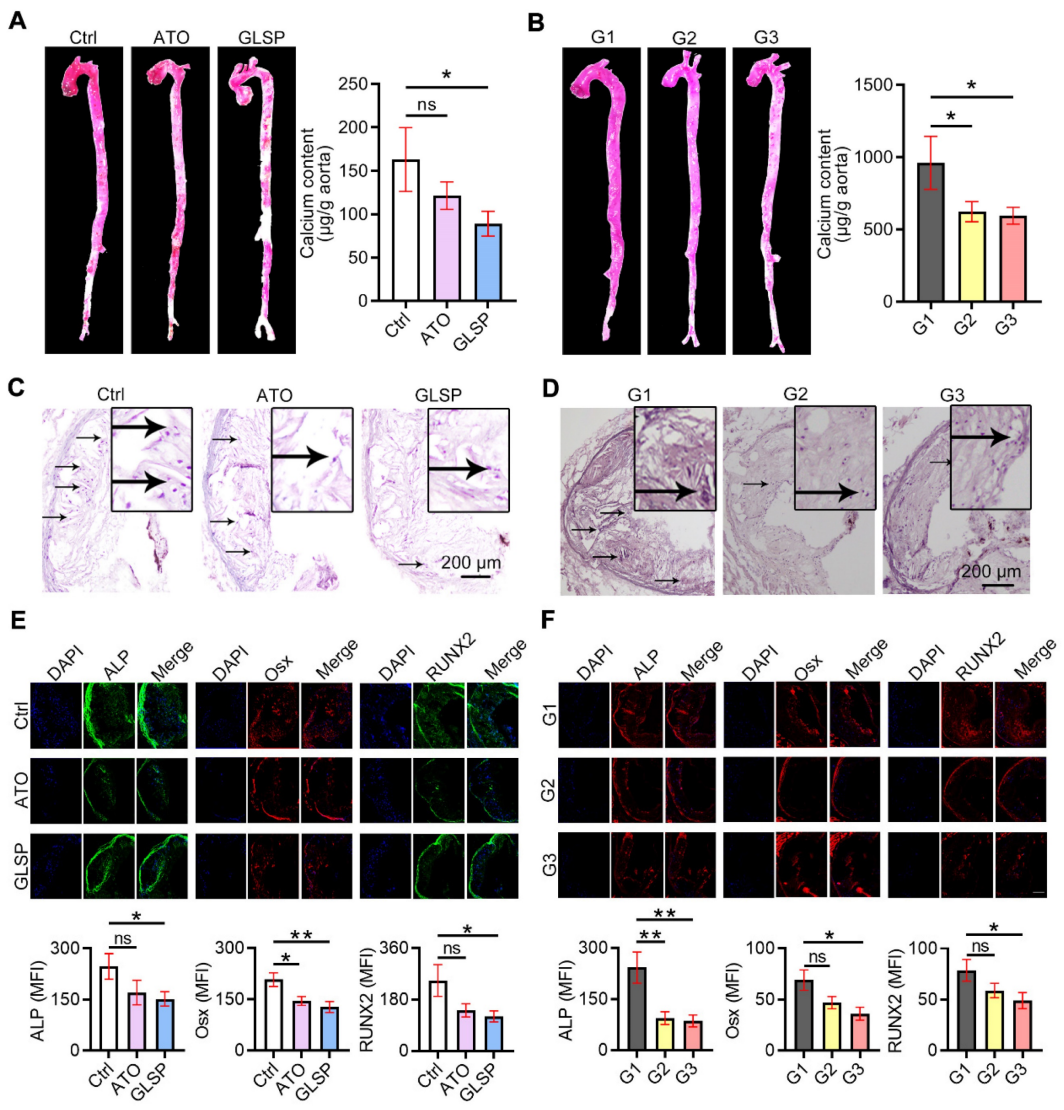
** GLSP inhibited vascular calcification.** (**A**) Alizarin red S staining of whole aorta in mice with early and advanced atherosclerosis. (**B**) Quantitative determination of calcium content in the aorta by calcium content assay kit in mice with early and advanced atherosclerosis, *P<0.05 compared with the Ctrl or G1 group. (**C**, **D**) Modified alizarin red S staining of aortic root cross-sections in mice with early and advanced atherosclerosis. (**E, F**) Serial frozen sections of the aortic root in mice with early and advanced atherosclerosis were used for immunofluorescence staining of ALP, Osx, and RUNX2, *P<0.05, **P<0.01 compared with the Ctrl or G1 group. All experiments are presented as mean ± SEM.

With the development of atherosclerosis, vascular calcification occurs under the regulation of calcification-related molecules. To explore the effects of GLSP on vascular calcification, we performed alizarin red S staining on the whole aorta and aortic root cross-sections as well as quantitative determination of calcium content in the aorta by calcium content assay kit. The results showed that calcification in the whole aorta, aortic root plaques, and calcium content in the whole aorta was significantly reduced after GLSP treatment (Figure 7A-D). In addition, to further explore the mechanism by which GLSP ameliorates aortic calcification, we performed immunofluorescence staining on the major regulators of calcification, including ALP, Osx, and RUNX2, in aortic root cross-sections. RUNX2 is the transcription factor activating osteoblast differentiation and ALP/BMP2 expression 31. Osx is also a downstream gene of RUNX2 32. The results showed that GLSP significantly downregulated the expression of ALP, Osx, and RUNX2 in plaques (Figure 7E-F), indicating that GLSP can inhibit arterial calcification by downregulating ALP, Osx, and RUNX2. Taken together, GLSP attenuates vascular calcification by inactivating the RUNX2 signaling pathway.

### The triterpenes from GLSP inhibited macrophage-derived foam cell formation and inflammation

**Figure 8:**
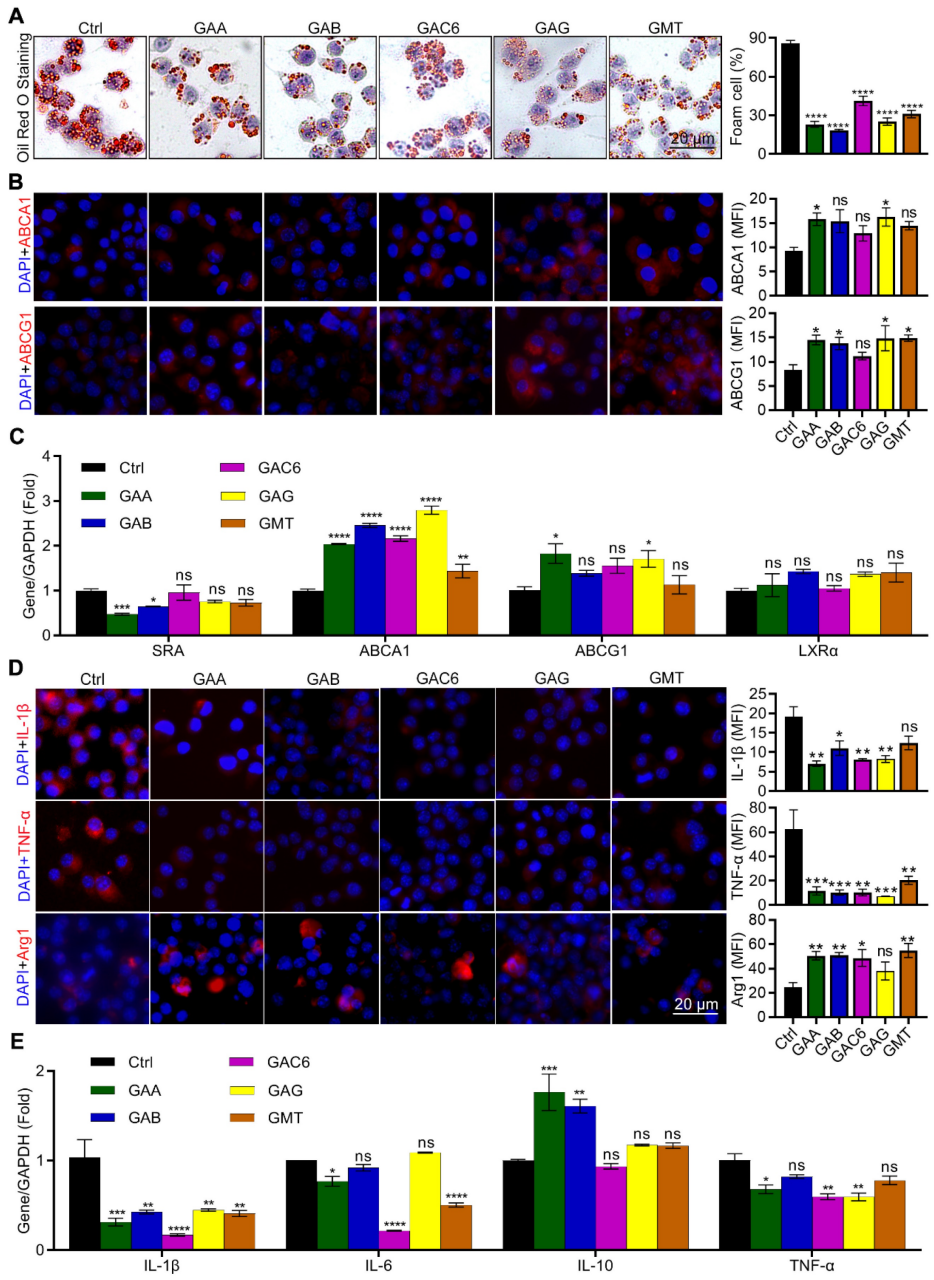
***Ganoderma lucidum* triterpenes improved lipid metabolism and exert anti-inflammatory effects.** (**A, B**) RAW264.7 cells were induced to be foam cell formation by ox-LDL in presence of the triterpenes, such as GAA (10 μM), GAB (10 μM), GAG (10 μM), GAC6 (10 μM), and GMT (10 μM), for 24 h. Effects of five triterpenes on the number of foam cells and the expression of ABCA1/ABCG1 *in vitro*. *P<0.05, ****P<0.0001 compared with the Ctrl group. (**C**) Effects of five triterpenes on the expression of genes responsible for lipid metabolism in RAW264.7 cells were determined by qRT-PCR. *P<0.05, **P<0.01, ***P<0.001, ****P<0.0001 compared with the Ctrl group. (**D**) RAW264.7 cells were induced inflammation by LPS in presence of the triterpenes, such as GAA (10 μM), GAB (10 μM), GAG (10 μM), GAC6 (10 μM), and GMT (10 μM), for 24 h. Effects of five triterpenes on the expression of inflammatory factors in cells, which was evaluated by immunofluorescent staining. *P<0.05, **P<0.01, ***P<0.001 compared with the Ctrl group. (**E**) Effects of five triterpenes on the gene expression of inflammatory factors in cells were determined by qRT-PCR. *P<0.05, **P<0.01, ***P<0.001, ****P<0.0001 compared with the Ctrl group. All experiments are presented as mean ± SEM.

Triterpenes are the major components of GLSP 33, 34. In this study, we identified thirteen triterpene components in GLSP by HPLC and measured their contents (Table S1, Figure S3). HPLC results indicated the five triterpenoids, including Ganoderic acid A (GAA), Ganoderic acid B (GAB), Ganoderic acid C6 (GAC6), Ganoderic acid G (GAG), and Ganodermanontriol (GMT). The cytotoxicity of these five triterpenoids was measured by CCK-8 assay and then these five triterpenoids were used for the *in vitro* experiments in determined concentrations (Figure S5). After treatment by triterpenoids, the oil red O and immunofluorescence staining were performed to determine their effects on lipid metabolism and inflammation in RAW264.7 cells. The results showed that five triterpenes of GLSP, including GAA, GAB, GAC6, GAG, and GMT, significantly reduced the number of foam cells (Figure 8A). Accordingly, GAA and GAG significantly upregulated the expression of ABCA1, and GAA, GAB, GAG, and GMT significantly upregulated the expression of ABCG1 in RAW264.7 cells (Figure 8B). In addition, the qRT-PCR results showed that all the above components significantly upregulated the expression of ABCA1, while GAA and GAG significantly upregulated the expression of ABCG1 (Figure 8C). To determine the anti-inflammatory effect of these components of GLSP, we incubated the RAW264.7 cells with the GLSP components respectively. Intriguingly, GAA, GAB, GAC6, and GAG significantly downregulated IL-1β and TNF-α expression and simultaneously upregulated Arg1 expression (Figure 8D). Accordingly, the qRT-PCR results showed that triterpenes from GLSP significantly downregulated the expression of IL-1β in RAW264.7 cells, and downregulated the expression of IL-6 and TNF-α to a different degree; GAA and GAB significantly upregulated the expression of IL-10. (Figure 8E). Taken together, the major components of GLSP, including GAA, GAG, GAC6, and GMT, could attenuate the foam cell formation and inflammation in macrophages, which may account for the protective effect of GLSP against atherogenesis.

### The triterpenes from GLSP inhibited VSMC osteogenic differentiation by inactivating RUNX2 expression and nuclear translocation

**Figure 9:**
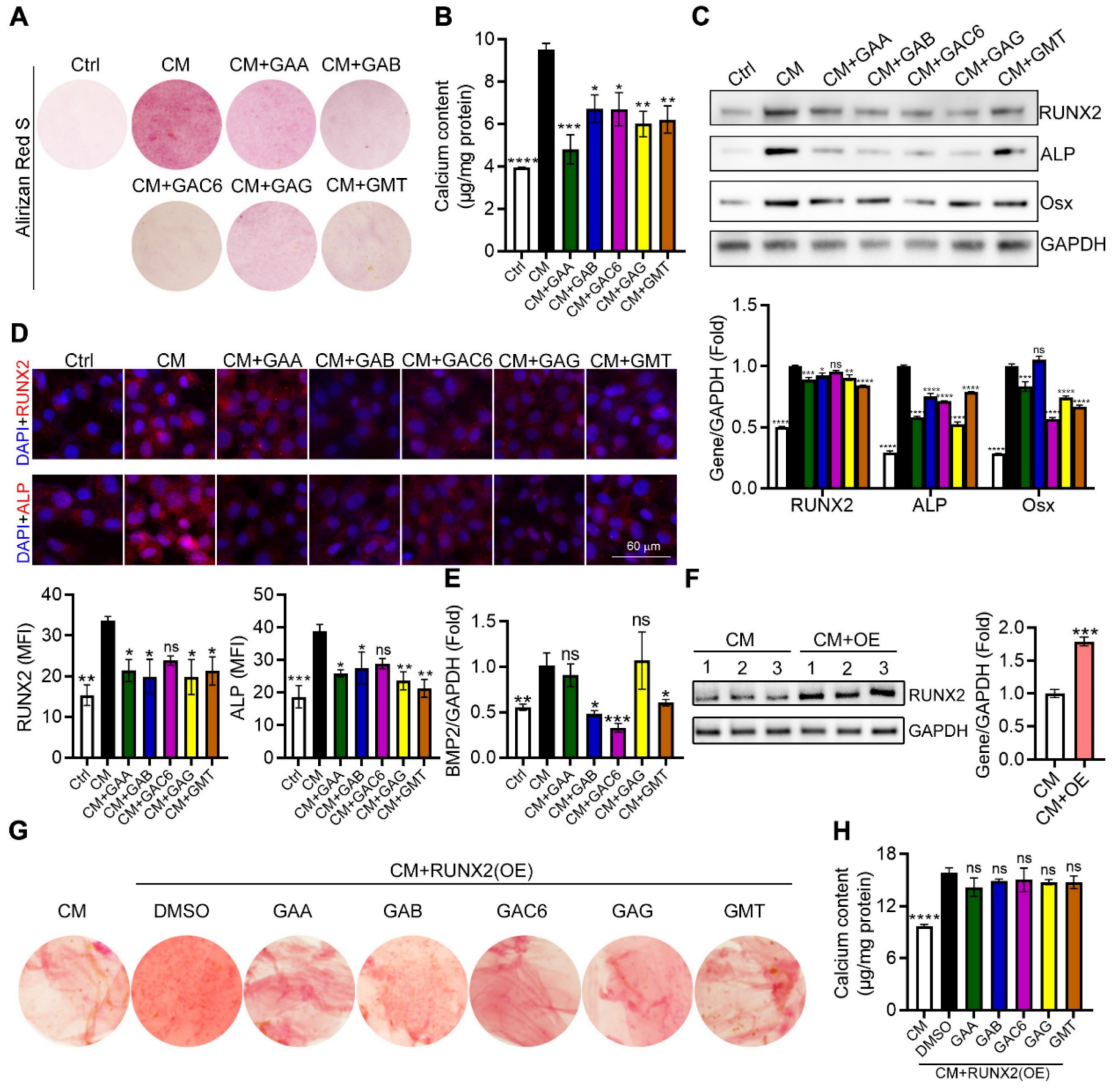
***Ganoderma lucidum* triterpenes inhibited calcification in HASMCs.** (**A**) HASMCs were induced calcification by incubation in CM in presence of the triterpenes, such as GAA (10 μM), GAB (10 μM), GAG (10 μM), GAC6 (10 μM), and GMT (10 μM), for 7 days. (**B**) Subsequently, calcium deposit by alizarin red S staining and calcium quantitative assay. *P < 0.05, **P<0.01, ***P<0.001, ****P<0.0001 compared with the CM group. (**C**) Expression of RUNX2, Osx, and ALP by Western blot. *P < 0.05, **P<0.01, ***P<0.001, ****P<0.0001 compared with the CM group. (**D**) The expression of RUNX2 and ALP by immunofluorescent staining of HASMCs with quantitation of RUNX2 and ALP MFI. *P < 0.05, **P<0.01, ***P<0.001 compared with the CM group. (**E**) Gene expression of BMP2 in HASMCs was determined by qRT-PCR. *P < 0.05, **P<0.01, ***P<0.001 compared with the CM group. (**F**) Expression of RUNX2 by Western blot in RUNX2-overexpressed HASMCs. ***P<0.001 compared with the CM group. (**G, H**) Calcium deposit was determined by alizarin red S staining and calcium quantitative assay in the RUNX2-overexpressed HASMCs. ****P<0.0001 compared with the DMSO group. All experiments are presented as mean ± SEM.

Calcium deposition in VSMCs significantly contributes to vascular calcification. To further determine the effects and the underlying mechanism of triterpenes from GLSP on VSMC calcification, calcium deposits in VSMC were induced by a calcification medium (CM) and determined by alizarin red S staining as well as calcium quantitative assay. We initially observed that treatment of HASMCs with triterpenes reduced cellular deposition of calcium (Figure 9A-B). In addition, we assessed the expression of vascular calcification markers, such as ALP and BMP2, and their upstream regulator RUNX2. RUNX2 is the transcription factor activating osteoblast differentiation and ALP/BMP2 expression 31. As the figure showed, CM induced the expression of ALP, Osx, and RUNX2 whereas triterpenes reduced ALP, Osx, and RUNX2 expression in HASMCs (Figure 9C). Furthermore, the result of the immunofluorescent staining showed that calcification-induced expression and nuclear translocation of RUNX2 and ALP were substantially attenuated by triterpenes (Figure 9D). Furthermore, the gene expression of BMP2 in HASMCs was measured by qRT-PCR, and the results showed that the osteogenic gene BMP2 was significantly downregulated in HASMCs under the treatment of triterpenes (Figure 9E). Next, the plasmid was transfected into HASMCs to induce RUNX2 overexpression, and the overexpression of RUNX2 in HASMCs was detected by Western blot (Figure 9F). GAA, GAB, GAC6, GAG, and GMT were administered to HASMC under the condition of overexpression of RUNX2, and the results showed that the inhibitory effect of GAA, GAB, GAC6, GAG, and GMT on calcium deposition and calcium content was markedly antagonized by RUNX2 overexpression in HASMCs (Figure 9G, H). Taken together, the major components of GLSP, including GAA, GAG, GAC6, and GMT, could attenuate VSMC calcification by inactivating RUNX2 expression, which partially accounts for the protective effect of GLSP against aortic calcification.

## Discussion

Cardiovascular disease remains the leading cause of death worldwide, which can increase the incidence of cardiovascular complications such as stroke, heart disease, and heart failure. Inflammation and lipid dysfunction are important contributors to both early and advanced atherosclerosis. Targeting inflammation and lipid metabolism are available strategies to treat atherosclerosis. Noticeably, during atherosclerosis development, vascular calcification always comes along and results in plaque vulnerability, which leads to the heavy risk of plaque rupture and the following cardiovascular event. However, at the present, no drug was available for treating vascular calcification during atherosclerosis development. In this study, we determined the protective effect of GLSP on atherosclerosis with calcification in LDLR^-/-^ mice and revealed the underlying mechanism *in vitro* experiments.

Foam cells, a characteristic feature of atherosclerosis which mainly mediated by hyperlipidemia, are also a major contributor and promoter of atherosclerotic plaque development 35, 36. Therefore, reducing the formation of foam cells or decreasing inflammatory factor levels induced by foam cells may be possible strategies to ameliorate atherosclerosis. ABCA1 and ABCG1, play a very important role in cholesterol efflux. Upregulation of their expression can promote cholesterol efflux and thus reduce foam cell formation to a certain extent 37, 38. For example, betulin can reduce atherosclerosis by upregulating ABCA1 and ABCG1 expression 39 or inhibiting the degradation of ABCA1^40^. Our study showed that GLSP can reduce lipid deposition in macrophage-derived foam cells, and then we examined the gene expression levels related to lipid production, consumption, and efflux, and found that GLSP can reduce lipid synthesis and increase lipid efflux, which mainly improves ABCA1/G1-mediated cholesterol efflux and further reducing atherosclerosis.

The liver is the terminal site of lipid metabolism and an important element in reverse cholesterol transport, which can metabolize lipids from circulation and cellular transport into bile acids for excretion to maintain lipid homeostasis 41. Accumulation of hepatic lipids can lead to hepatic steatosis, which can lead to decreased hepatic lipid metabolism and more lipid deposition in blood vessels and cells, leading to atherosclerosis. Therefore, reducing lipid accumulation in the liver and liver steatosis, and maintaining the normal physiological structure of the liver is a prerequisite for the liver to perform the function of lipid metabolism, and can inhibit atherosclerosis to a certain extent 29. Our study revealed that GLSP could downregulate genes of lipid synthesis and upregulate genes of lipolysis in the liver, by which reducing hepatic lipid accumulation and steatosis.

Calcification is an important contributor to atherosclerotic plaque rupture. Calcification is divided into media calcification and intimal calcification, which are involved by VSMC, and the former predominates in the atherosclerotic process. VSMC change from a contractile phenotype to an osteo/chondrocyte-like phenotype in response to inflammatory factors, oxidative stress, and mechanical stimuli 42, 43. This phenotypic shift is accompanied by a change in markers, such as decreased expression of smooth muscle cell markers SM22α, α-SMA, and increased expression of bone/chondrogenic markers RUNX2, and osteopontin 42. RUNX2 is a key transcription factor in the regulation of VSMC osteogenic differentiation and calcification. BMP2, ERK/MAPK, and PI3K/AKT signaling pathways induce RUNX2 expression in VSMC, promoting vascular calcification and atherosclerosis while pharmacological inhibition or degradation of RNUX2 can reduce calcification. For instance, microRNA-34a could reduce vascular calcification through the NOTCH1-RUNX2 signaling pathway 44. In this study, alizarin red S staining of the aortic root and measurement of aortic calcium content showed a significant reduction in calcification after GLSP treatment. Moreover, the osteogenic genes, including RUNX2, Osx, and ALP, were found to decrease significantly, suggesting that GLSP can significantly improve atherosclerotic calcification.

To determine the triterpenes from GLSP on atherosclerosis and vascular calcification. We determined the major triterpenes of GLSP by HPLC, including Ganoderic acid A, Ganoderic acid B, Ganoderic acid C6, Ganoderic acid G, and Ganodermanontriol. Subsequently, these components were utilized to incubate the macrophages and VSMC to assess the inhibiting effect on foam cell formation, inflammation, and VSMC osteogenic differentiation and calcification. In line with the results of *in vivo* experiments, these components inhibited foam cell formation by enhancing ABCA1/G1-mediated cholesterol efflux and attenuated calcification by inactivating RUNX2-mediated osteogenesis. Taken together, the anti-atherosclerotic effects of GLSP and its triterpenoids were achieved by enhancing ABCA1/G1-mediated cholesterol efflux and Inhibiting RUNX2-mediated osteogenesis.

In this study, we determined the protective effect of GLSP against atherosclerosis and vascular calcification in LDLR^-/-^ mice, which may be attributed to inhibiting inflammation and apoptosis in plaque; and reducing lipid accumulation in plaque and liver. Mechanistically, these pharmacological properties of GLSP were through enhancing ABCA1/G1-mediated cholesterol efflux and reducing RUNX2-mediated VSMC osteogenic differentiation and calcification. At present, there is no effective treatment for vascular calcification during atherosclerosis development clinically. Therefore, GLSP may act as a novel therapeutic strategy for treating atherosclerosis and vascular calcification.

## Methods

### Reagents

Antibodies for Arg1 (Cat#: ab212522), CD31 (Cat#: ab28364) and ABCG1 (Cat#: ab52617) were purchased from Abcam (Cambridge, MA). Antibodies for IL-1β (Cat#: sc-52012), ICAM-1 (Cat#: sc-107), VCAM-1 (Cat#: sc-13160), ALP (Cat#: sc-365765), Οsx (Cat#: sc-393325) and RUNX2 (Cat#: sc-390351) were purchased from Santa Cruz Biotechnology (Santa Cruz, CA). Antibodies for Cleaved Caspase1 (Cat#: 4199), ABCA1 (Cat#: 96292), β-actin (Cat#:4970) were purchased from Cell Signaling Technology (Danvers, MA). TG assay kit (Cat#:100020090), total cholesterol assay kit (Cat#: 100020080), LDL-C assay kit (Cat#:100020245), HDL-C assay kit (Cat#:100020235) were purchased from Biosino Bio-Technology and Science INC (Beijing, China). Alizarin Red S solution (Cat#: G3280) was purchased from Solarbio & Technology Co., Ltd (Beijing, China). IL-1β Elisa kit (Cat#: SEA563Mu), IL-6 Elisa kit (Cat#: SEA079Mu), TNF-α Elisa kit (Cat#: SEA133Mu), IL-10 Elisa kit (Cat#: SEA056Mu) and SOD Elisa kit (Cat#: SES134Mu) were purchased from Cloud-Clone Corp. (Wuhan, China). Mouse ROS Elisa kit (Cat#: YX-181519M) and Mouse ox-LDL Elisa kit (Cat#: YX-152412M) were purchased from Sino Best Biological Technology CO., Ltd (Shanghai, China). Calcium Colorimetric Assay kit (Cat#: MAK022-1KT) was purchased from Sigma-Aldrich. All other reagents were purchased from Sigma-Aldrich except where indicated.

### Cell culture

RAW264.7 cells and HASMCs were cultured in this study. The frozen cells were taken out from the liquid nitrogen and transferred to the 37 °C warm water bath quickly for about 1 min; the resuscitated cells were then placed into DMEM/F12 or RPMI 1640 medium containing 10% fetal bovine serum and 50 μg/mL penicillin/streptomycin and 2 mM glutamine. The suspended cells were transferred to a centrifuge tube for centrifugation at 800 rpm for 10 min, and the supernatant culture medium was removed. DMEM/F12 or RPMI 1640 medium was added, and the cells were blown gently and distributed to culture dish, then cells were incubated in the cell incubator at 37 °C with 0.5% CO_2_. The drugs were administered when the cell density reached more than 70%. The cells were slightly rinsed with sterile PBS, and then the serum-free DEME medium was added. Subsequently, the drugs prepared with DMSO were added into the medium at the indicated concentration to incubate the cells.

### Animal studies

All animal care and experimental protocols for in *vivo* studies conformed to the Guide for the Care and Use of Laboratory Animals published by the NIH (NIH publication no. 85-23, revised 1996) and approved by First Teaching Hospital of Tianjin University of Traditional Chinese Medicine. Male LDLR^-/-^mice (8 weeks old) were purchased from the Changzhou Kavens Laboratory Animal Co. Ltd. (Nanjing, Jiangsu, China). These mice were maintained at the Animal Center of Chu Hsien-I Memorial Hospital with free access to food and water. Mice were allowed to acclimatize to their housing environment for at least 7 days before experiments. LDLR^-/-^ mice were randomly divided into 3 groups: Ctrl group, ATO group, and GLSP group, in which atorvastatin was used as a positive drug and GLSP was used as a treatment drug. The atherosclerosis model was induced by feeding a high-fat diet (HFD, 21% fat, 0.5% cholesterol, MD12015HL, medicience Ltd.). Based on the formula for conversion of body surface area between mice and humans, the dose of atorvastatin (Pfizer Pharmaceuticals Ltd.) used in mice was converted as 4 mg/day/kg body weight, and the dose of GLSP (Zhejiang ShouXianGu Botanical Drug Institute) was converted as 1400 mg/day/kg body weight. Calculated drugs were mixed with the high-fat diet. The mice were fed HFD continuously for 12 weeks in the early atherosclerosis model and 36 weeks in the advanced atherosclerosis model, respectively. The treatment during the experiment was conducted blindly. During the treatment, the animals were checked daily for intake of food and water, and weekly for their body weight.

### Foam cells detection

Three mice in each group were randomly selected to extract the abdominal macrophages. Briefly, each mouse was injected intraperitoneally with 3 mL of 4% sulfur gelatin. 4 days later, mice were sacrificed and 10 mL sterile PBS was injected intraperitoneally. The PBS containing macrophages was extracted with a syringe and carefully rinsed repeatedly. The extracted liquid was pumped into a 15 mL centrifuge tube, and red blood cell lysis buffer was added to remove red blood cells. The cells were centrifuged at 800 rpm for 10 min and the supernatant was discarded. The complete RPMI 1640 medium containing 10% FBS and 50 μg/mL penicillin/streptomycin was added into the centrifuge tube to resuspend cells, and cells were seeded in 24-well plates. After 1 day, the adherent cells were washed gently with PBS, and the pre-filtered oil red O solution was added to each well for 1 h. Subsequently, the oil red O solution was discarded and hematoxylin solution was added to each well for 10 min. Cells were photographed under a 40× visual field eventually and foam cells were counted as previously described 45.

### Lesion detection by Oil Red O staining

The lesion was indicated by the lipid content in the whole aorta and aortic arch which was determined by oil red O staining. Briefly, the aorta of mice was separated and the excess tissue was stripped. Before staining, the oil red O solution was filtered. The aorta was stained with oil red O solution for 1 h. Photographs were subsequently taken under a stereomicroscope. Frozen sections of the aortic root were also stained by oil red O solution for 1 h and were photographed with a microscope.

### HE staining, Sirius red staining, and immunofluorescent staining

The frozen sections of the aortic root were stained with hematoxylin staining for 2 min and gently washed with running water, then the frozen sections were stained with eosin staining for 30 s, and the necrotic core areas in the plaques were counted to evaluate plaque stability. Moreover, the frozen sections of the aortic root were stained with sirius red staining for 1 h, then stained with hematoxylin staining for 10 min, and collagen areas in plaques were counted to evaluate plaque stability. The frozen sections of the aortic root were stained with immunofluorescent staining. Briefly, the frozen sections of aortic roots were sealed with 2% BSA for 1 h, then a diluted primary antibody was added and placed in the refrigerator at 4 °C overnight. The next day, the frozen sections were washed three times with PBS for 10 min each time and incubated with secondary antibody for 1 h at 37 °C. Then the frozen sections were washed three times with PBS again and sealed in the dark. Subsequently, the fluorescence intensity of inflammatory cytokine and calcification-related molecules was measured by a fluorescence microscope.

### Determination of calcification *in vivo* and *in vitro*

Vascular calcification formed *in vivo* or *in vitro* was determined by alizarin red S staining of aortic root cross sections and calcium quantitative assay. Briefly, the frozen sections of aortic roots were stained with alizarin red S for 5 min, differentiated with McGee-Russell differentiation solution for several seconds, and then stained with hematoxylin for 2 min. The calcium quantitative assay was performed according to the steps in the kit instructions. *In vitro*, HASMCs were induced calcification by culture in complete DMEM/F12 medium (1:1) containing 5 mM Pi (mixture of NaH_2_PO_4_ and Na_2_HPO_4_, ratio 1:2, pH7.4) and 50 μg/mL ascorbic acid or plus treatment for 7 days, followed by alizarin red S staining and calcium quantitative assay 46, 47.

### Western blot and qRT-PCR analysis

The total protein of cells or tissues was extracted, then the protein concentration was measured by the BCA protein quantification kit, and the protein was quantified to 1 μg/μL. Subsequently, protein expression of ABCA1, ABCG1, and β-actin was determined by Western blot. Total RNA of cells or tissues was extracted by RNA extraction assay kit, the concentration of RNA was measured and mRNA was reverse transcribed to cDNA, next, primers and SYBR mix were added to detect the gene expression of eNOS, TGFβ, Arg1, IL-1β, IL-10, SRA, ABCA1, ABCG1, HMGCR, FASN, CPT1α, ACC1, ATGL, HSL, SREBP1, SCD1, PPARγ, IL-6, TNF-α. Primer sequences were shown in supplementary data table S2. All reactions were performed three times.

### Determination of triterpenes in GLSP by HPLC

Ganoderic acid I, Ganoderic acid C2, Ganoderic acid C6, Ganoderic acid G, Ganoderic acid B, Ganoderic acid N, Ganoderic acid B, Ganoderic acid A, Ganoderic acid H, Ganoderic acid D2, Ganoderic acid D, Ganoderic acid C1, and Ganodermanontriol control substance were weighed with 10mg, and then dissolved in methanol to make the working solution. The corresponding concentration was 10.24 mg/mL, 19.78 mg/mL, 10.33 mg/mL, 16.29 mg/mL, 5.02 mg/mL, 4.66 mg/mL, 21.15 mg/mL, 21.18 mg/mL, 18.22 mg/mL, 8.63 mg/mL, 5.56 mg/mL, 14.42 mg/mL and 9.68 mg/mL. Subsequently, the testing solution was prepared for testing. Briefly, 1.0 g GLSP was dissolved in 40 mL methanol for ultrasonic extraction for 30 min and filtered by 0.22 mm microporous membrane for liquid chromatograph determination. The determination was performed on a chromatographic column (Waters CORTECS T3, 4.6 mm×150 mm, 2.7 mm). Mobile phase A was 0.1% formic acid aqueous solution, mobile phase B was acetonitrile, and the gradient elution condition was 0-26 min, 25.0%-25.5% B; 26-29 min, 25.5%-30% B; 29-34 min, 30%-30% B; 34-40 min, 30%-40% B; 40-54 min, 40%-70% B; 54-55 min, 70%-100% B; 55-62 min, 100%-100% B. The flow rate was 1.0 mL/min; the column temperature was 40 °C; the detection wavelength was 254 nm, and the injection volume was 5 mL.

### Plasmid transfection for RUNX2 expression

Cells were seeded to a 24-well plate. On the day of transfection, for each well of cells, 0.5 mg DNA was diluted in 50 mL serum-free medium and 1.5 mL liposomal nucleic acid transfection reagent was diluted in 50 mL serum-free medium. Diluted DNA and diluted liposomal nucleic acid transfection reagent were mixed, gently blended, and incubated at room temperature for 20 min to form DNA-liposome complexes. Subsequently, 100 mL of the complex was added to each well of the cell culture plate, and the plate was shaken and gently mixed. The 24-well plates were incubated for 48 h in a 37 °C, 5% CO_2_ incubator.

### Statistical analysis

The data and statistical analysis comply with the recommendations on experimental design and analysis in pharmacology. All data are expressed as mean ± SEM or mean ± SD. An unpaired Student's t test was used for comparisons between two groups, or One-way ANOVA for comparisons between multiple groups followed by Turkey's method. Significance was accepted when P < 0.05.

## Supplementary Material

Supplementary figures and tables.

## Abbreviations

GLSP: *Ganoderma lucidum* spore powder
GLT: *Ganoderma lucidum* triterpenes
ABCA1/G1: ATP-binding cassette transporter A1/G1
VSMC: vascular smooth muscle cell
HFD: high-fat diet
ICAM-1: intercellular adhesion molecule-1
VCAM-1: vascular cell adhesion molecule-1
GAA: Ganoderic acid A
GAB: Ganoderic acid B
GAC6: Ganoderic acid C6
GAG: Ganoderic acid G
GMT: Ganodermanontriol
HDL: high-density lipoprotein
ox-LDL: oxidized low-density lipoprotein
LPS: lipopolysaccharide
CM: calcification medium
ATO: atorvastatin
