# Supplementary material for: GLSP and GLSP-derived triterpenes attenuate atherosclerosis and aortic calcification by stimulating ABCA1/G1-mediated macrophage cholesterol efflux and inactivating RUNX2-mediated VSMC osteogenesis
Source: Theranostics. 2023 Feb 21;13(4):1325–41. doi: 10.7150/thno.80250 (PMC10008734; doi:10.7150/thno.80250)
Supplement: Supplementary file 1 — Supplementary figures and tables. [file thnov13p1325s1.pdf]

GLSP and it-derived triterpenes attenuate atherosclerosis and aortic calcification by improving ABCA1/G1-mediated cholesterol efflux and inactivating RUNX2-mediated osteogenesis

Guobin Zheng[3#], Yun Zhao[1,2#], Zhenhao Li[5#], Yunqing Hua[1,2], Jing Zhang[1,2], Yaodong Miao[6], Yang Guo[2], Lan Li[1,2], Jia Shi[1,2], Zhengwei Dong[1,2], Shu Yang[4*], Guanwei Fan[1,2*], Chuanrui Ma[1,2*]

[1]First Teaching Hospital of Tianjin University of Traditional Chinese Medicine, Tianjin, China

[2]National Clinical Research Center for Chinese Medicine Acupuncture and Moxibustion, Tianjin, China

[3]NHC Key Laboratory of Hormones and Development, Tianjin Key Laboratory of Metabolic Diseases, Chu Hsien-I Memorial Hospital & Tianjin Institute of Endocrinology, Tianjin Medical University, Tianjin 300134, China

[4]Department of Geriatrics, The Second Clinical Medical College, Jinan University (Shenzhen People's Hospital), Shenzhen, China

[5]Zhejiang ShouXianGu Botanical Drug Institute, Zhejiang Hangzhou 321200 China

[6]Second Affiliated Hospital of Tianjin University of Traditional Chinese Medicine, Tianjin, P. R. China

#These authors contributed equally to this article.

Correspondence should be addressed to:

Chuanrui Ma, PhD；Guanwei Fan, PhD；Shu Yang, PhD

First Teaching Hospital of Tianjin University of Traditional Chinese Medicine, National Clinical Research Center for Chinese Medicine Acupuncture and Moxibustion. No.88, Chang Ling Road, Li Qi Zhuang Jie, Xi Qing District, Tianjin, P.R. China;

E-mail: chuanruima2013@mail.nankai.edu.cn; guanwei.fan@tjutcm.edu.cn; yang.shu@szhospital.com

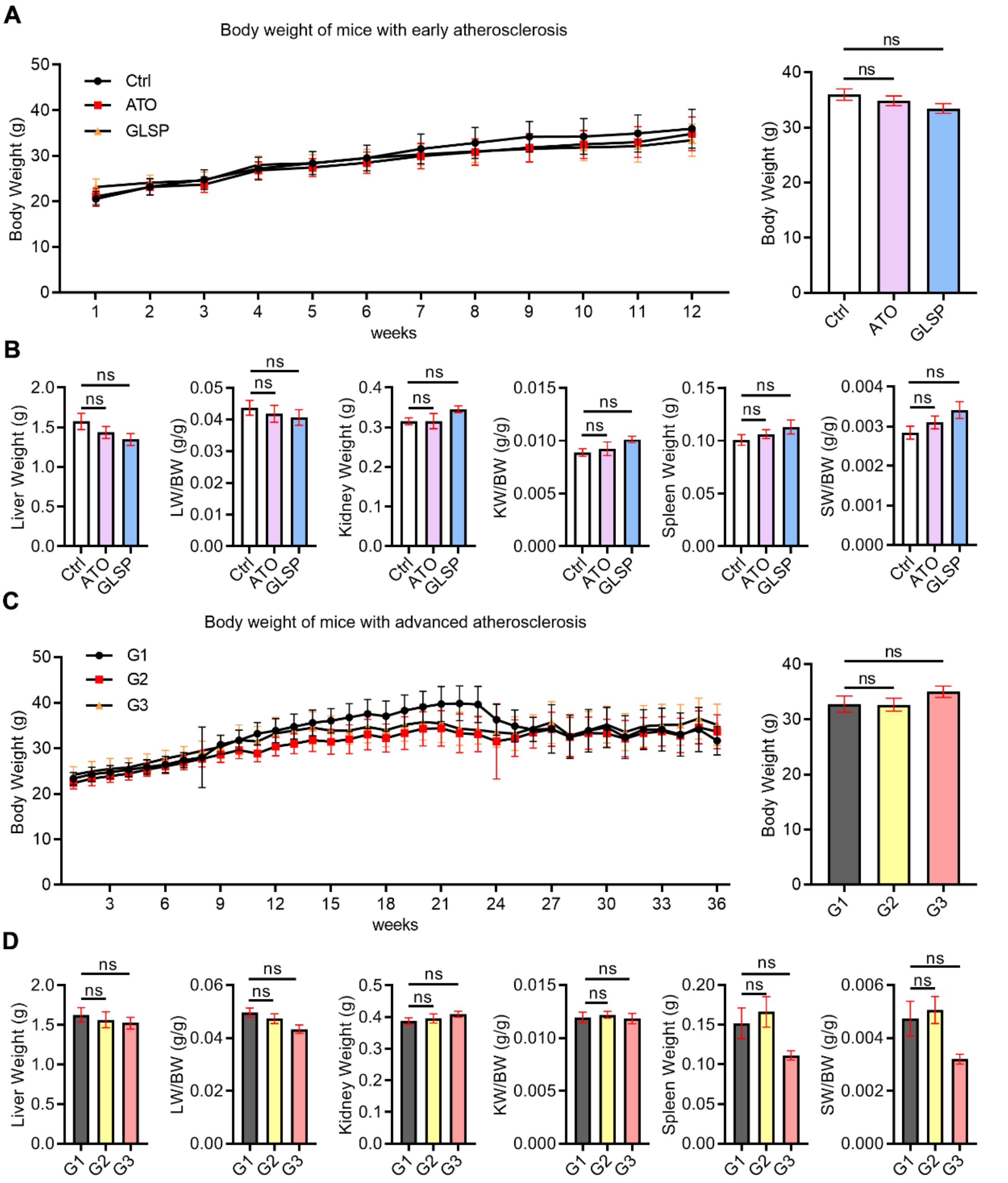

Figure S1. Body and organ weights of early and advanced LDLR$^{-/-}$ mice. (**A**) Early LDLR$^{-/-}$ mice weight change curve, endpoint weight and endpoint change difference. (**B**) Liver，kidney and spleen weights，and liver-to-body, kidney-to-body ratios and spleen-to-body ratios in early LDLR$^{-/-}$ mice. (**C**) Advanced LDLR$^{-/-}$ mice weight change curve, endpoint weight and endpoint change difference. (**D**) Liver，kidney and spleen weights，and liver-to-body, kidney-to-body ratios and spleen-to-body ratios in advanced LDLR$^{-/-}$ mice. *P<0.05, **P<0.01, ***P<0.001, ****P<0.0001 compared with the Ctrl or the G1 group.

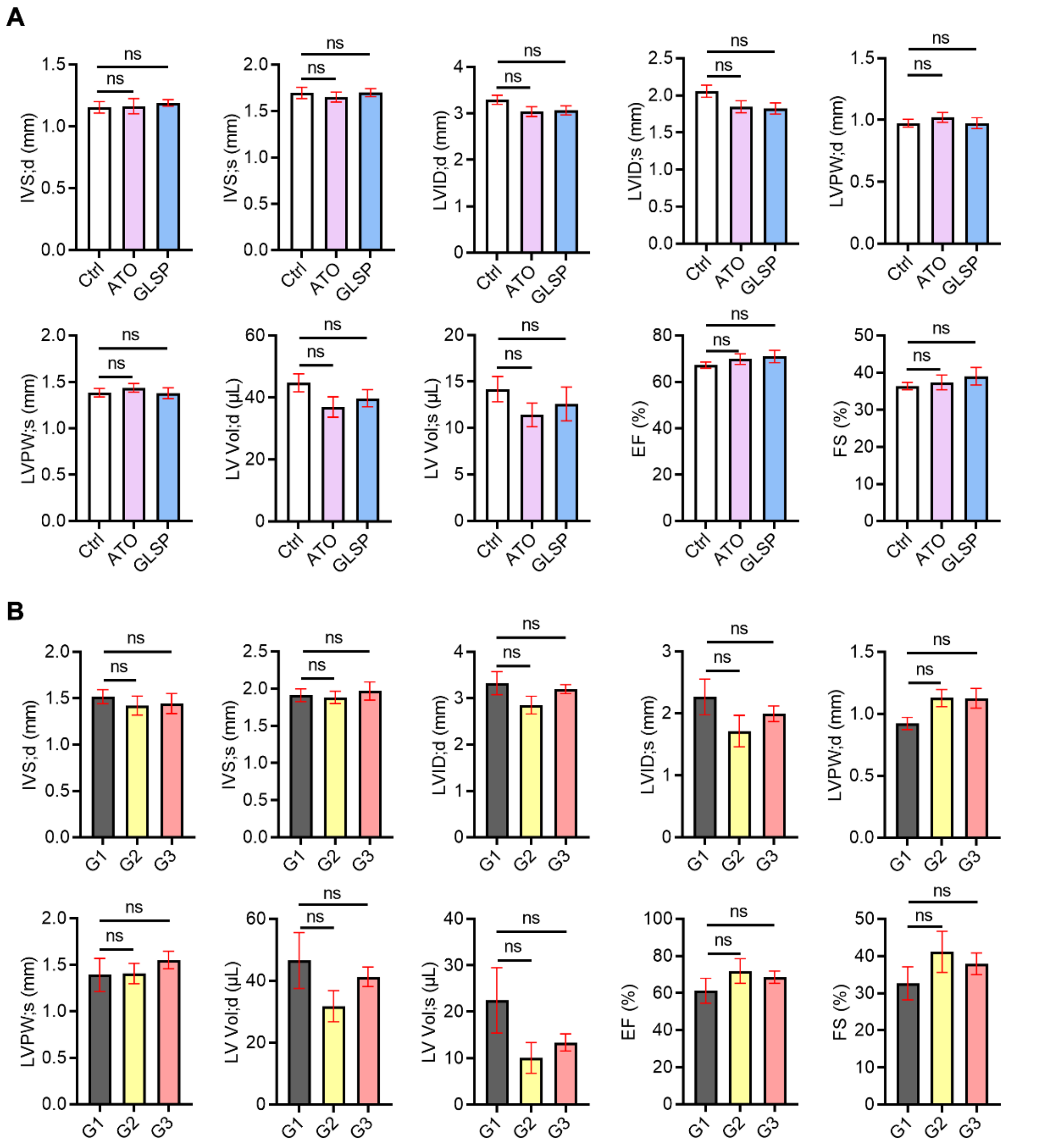

Figure S2. Cardiac ultrasound data in early and advanced LDLR$^{-/-}$ mice. (**A**) Cardiac ultrasound data in early LDLR$^{-/-}$ mice. (**B**) Cardiac ultrasound data in advanced LDLR$^{-/-}$ mice.

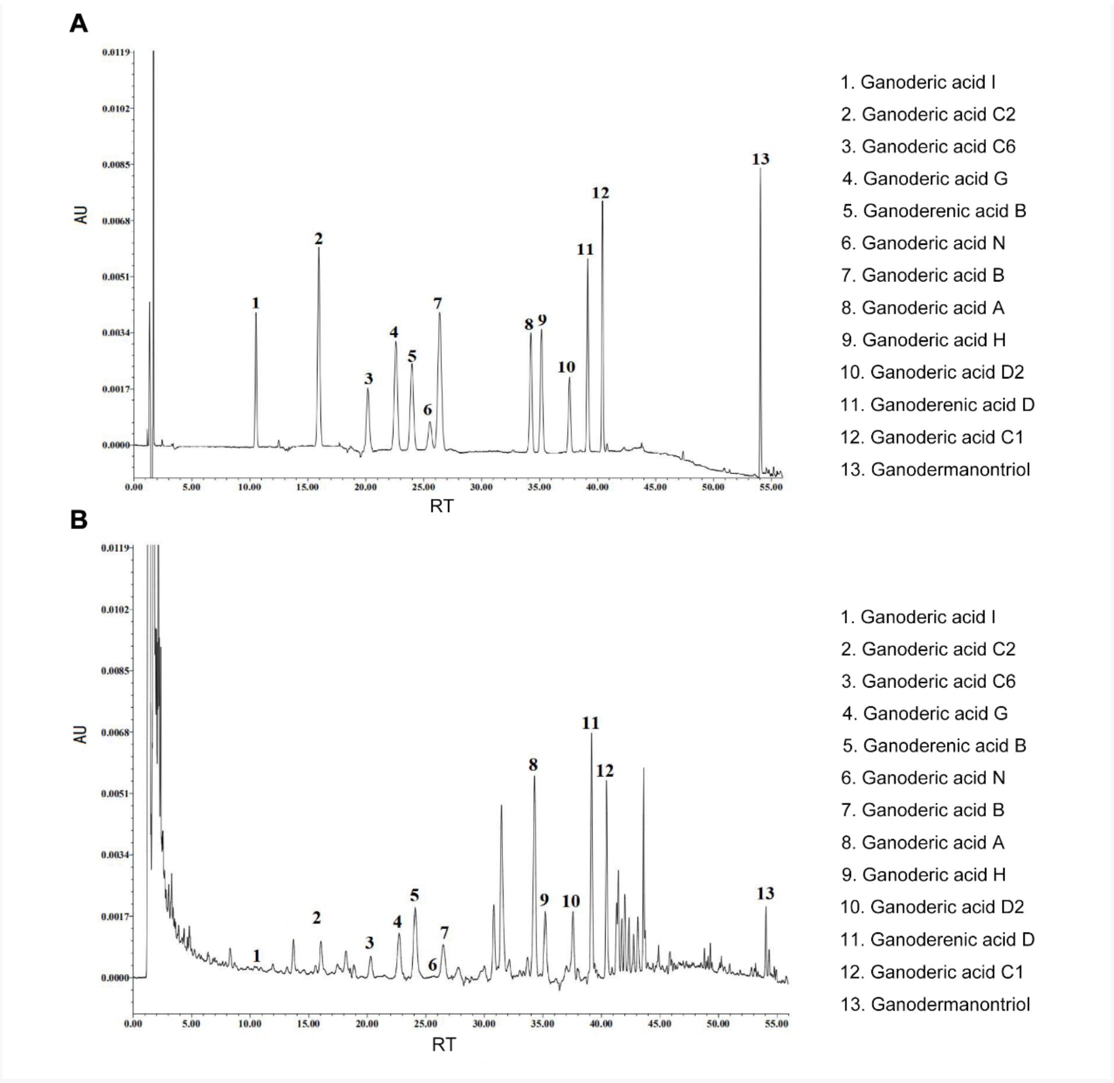

Figure S3. Chromatogram of thirteen triterpene components in GLSP by HPLC. (**A**) Chromatogram of standard. (**B**) Chromatogram of sample.

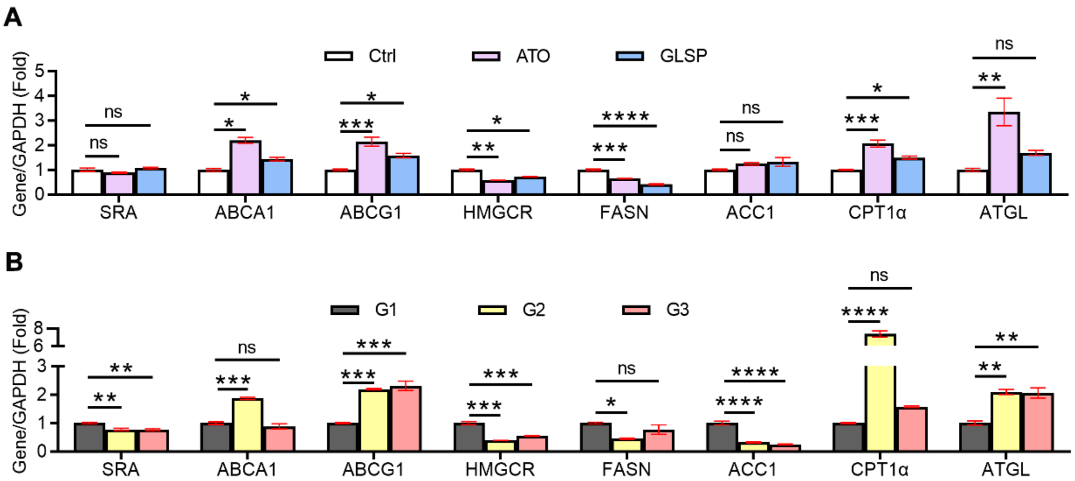

Figure S4. Effects of GLSP on the expression of genes related to lipid metabolism in LDLR$^{-/-}$ mice peritoneal macrophages. (**A**) Effects of GLSP on the expression of genes related to lipid metabolism in early LDLR$^{-/-}$ mice peritoneal macrophages. (**B**) Effects of GLSP on the expression of genes related to lipid metabolism in advanced LDLR$^{-/-}$ mice peritoneal macrophages. *P<0.05, **P<0.01, ***P<0.001, ****P<0.0001 compared with the Ctrl or G1 group.

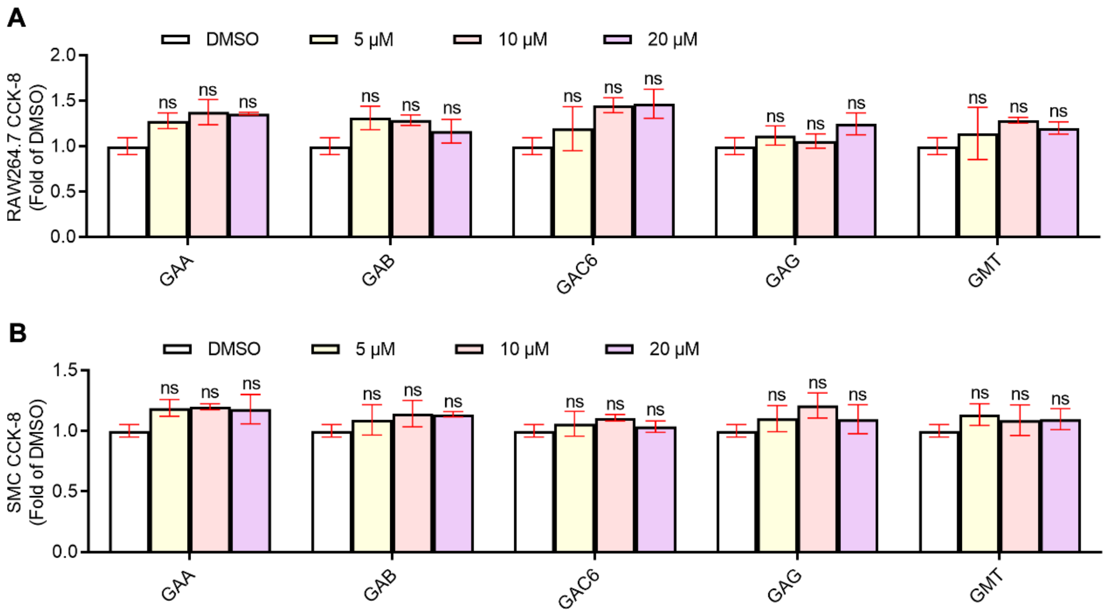

Figure S5. Cell viability of RAW264.7 and HASMC was determined by CCK-8 assay. (**A**) $4x10^3$ RAW264.7 cells were seeded in 96-well plates，GAA, GAB, GAC6, GAG and GMT (5, 10, 20 μM) were administered and cell viability was measured after 16 h. (**B**) $4x10^3$ HASMCs were seeded in 96-well plates，GAA, GAB, GAC6, GAG and GMT (5, 10, 20 μM) were administered and cell viability was measured after 16 h. ns: no significance.

Table S1. Identification and content determination of thirteen triterpene components in Ganoderma lucidum spore powder

Number	Compound name	Content (%)
1	Ganoderic acid I	0.004
2	Ganoderic acid C2	0.026
3	Ganoderic acid C6	0.026
4	Ganoderic acid G	0.058
5	Ganoderenic acid B	0.036
6	Ganoderic acid N	0.002
7	Ganoderic acid B	0.044
8	Ganoderic acid A	0.338
9	Ganoderic acid H	0.100
10	Ganoderic acid D2	0.094
11	Ganoderenic acid D	0.070
12	Ganoderic acid C1	0.112
13	Ganodermanontriol	0.022
Gross weight		0.932

Table S2. The sequences of primers for qRT-PCR analysis

Gene	Forward	Backward
Mus GAPDH	AGGTCGGTGTGAACGGATTTG	TGTAGACCATGTAGTTGAGGTCA
Mus eNOS	GGCTGGGTTTAGGGCTGTG	CTGAGGGTGTCGTAGGTGATG
Mus IL-1β	GCAACTGTTCCTGAACTCAACT	ATCTTTTGGGGTCCGTCAACT
Mus IL-10	GCTCTTACTGACTGGCATGAG	CGCAGCTCTAGGAGCATGTG
Mus Arg1	CTCCAAGCCAAAGTCCTTAGAG	AGGAGCTGTCATTAGGGACATC
Mus TGFβ	CTCCCGTGGCTTCTAGTGC	GCCTTAGTTTGGACAGGATCTG
Mus SRA	GCACAATCTGTGATGATCGCT	CCCAGCATCTTCTGAATGTGAA
Mus LXRα	ATGTCTTCCCCCACAAGTTCT	GACCACGATGTAGGCAGAGC
Mus ABCA1	AAAACCGCAGACATCCTTCAG	CATACCGAAACTCGTTCACCC
Mus ABCG1	CTTTCCTACTCTGTACCCGAGG	CGGGGCATTCCATTGATAAGG
Mus HMGCR	TGTTCACCGGCAACAACAAGA	CCGCGTTATCGTCAGGATGA
Mus FASN	GGCTCTATGGATTACCCAAGC	CCAGTGTTCGTTCCTCGGA
Mus ACC1	CTCCCGATTCATAATTGGGTCTG	TCGACCTTGTTTTACTAGGTGC
Mus CPT1α	AGATCAATCGGACCCTAGACAC	CAGCGAGTAGCGCATAGTCA
Mus ATGL	CTGAGAATCACCATTCCCACATC	CACAGCATGTAAGGGGGAGA
Mus SREBP1	GATGTGCGAACTGGACACAG	CATAGGGGGCGTCAAACAG
Mus SCD1	TTCTTGCGATACACTCTGGTGC	CGGGATTGAATGTTCTTGTCGT
Mus HSL	TGGCACACCATTTTGACCTG	TTGCGGTTAGAAGCCACATAG
Mus PPARγ	GGAAGACCACTCGCATTCCTT	GTAATCAGCAACCATTGGGTCA
